# AFCLNet: An Attention and Feature-Consistency-Loss-Based Multi-Task Learning Network for Affective Matching Prediction in Music–Video Clips

**DOI:** 10.3390/s26010123

**Published:** 2025-12-24

**Authors:** Zhibin Su, Jinyu Liu, Luyue Zhang, Yiming Feng, Hui Ren

**Affiliations:** 1 State Key Laboratory of Media Convergence and Communication, Beijing 100024, China; suben@cuc.edu.cn; 2 Key Laboratory of Acoustic Visual Technology and Intelligent Control System, Ministry of Culture and Tourism, Beijing 100024, China; ljy_123@cuc.edu.cn (J.L.); zhangluyue@cuc.edu.cn (L.Z.); fyming8888@163.com (Y.F.); 3 School of Information and Communication Engineering, Communication University of China, Beijing 100024, China

**Keywords:** audiovisual affective matching, DCCA, attention, feature-consistency loss

## Abstract

Emotion matching prediction between music and video segments is essential for intelligent mobile sensing systems, where multimodal affective cues collected from smart devices must be jointly analyzed for context-aware media understanding. However, traditional approaches relying on single-modality feature extraction struggle to capture complex cross-modal dependencies, resulting in a gap between low-level audiovisual signals and high-level affective semantics. To address these challenges, a dual-driven framework that integrates perceptual characteristics with objective feature representations is proposed for audiovisual affective matching prediction. The framework incorporates fine-grained affective states of audiovisual data to better characterize cross-modal correlations from an emotional distribution perspective. Moreover, a decoupled Deep Canonical Correlation Analysis approach is developed, incorporating discriminative sample-pairing criteria (matched/mismatched data discrimination) and separate modality-specific component extractors, which dynamically refine the feature projection space. To further enhance multimodal feature interaction, an Attention and Feature-Consistency-Loss-Based Multi-Task Learning Network is proposed. In addition, a feature-consistency loss function is introduced to impose joint constraints across dual semantic embeddings, ensuring both affective consistency and matching accuracy. Experiments on a self-collected benchmark dataset demonstrate that the proposed method achieves a mean absolute error of 0.109 in music–video matching score prediction, significantly outperforming existing approaches.

## 1. Introduction

With the rapid development of smart mobile sensing technologies, modern devices are increasingly capable of capturing fine-grained audiovisual signals in real time, making music–video emotion matching prediction an essential task for affective multimedia analysis and context-aware intelligent services. For example, in video editing, the automated alignment of scenes with emotionally congruent background music can significantly reduce post-production costs. On short-form video platforms, dynamic music recommendation systems leverage affective adaptation to enhance user retention. In immersive environments, maintaining affective consistency across modalities optimizes human–computer interaction. Nevertheless, the inherent heterogeneity of music and video data, combined with the complexity of affective expression, poses substantial challenges: existing methods often struggle with feature-alignment difficulties, affective semantic gaps, and decoupling biases.

Early research on music–video matching mainly relied on unimodal features for cross-modal alignment. For example, the authors of a previous study [1] employed predefined affective labels to retrieve background music; however, these annotations were constrained by label sparsity and subjective bias. Similarly, TuneSensor [2] applied semantic data mining to extract multimodal association rules, but this method overlooked dynamic affective interactions, resulting in a cognitive gap between low-level signals and high-level semantics. The CMRA framework [3] employed traditional classifiers on handcrafted features, but their modeling capacity was insufficient to capture complex, nonlinear cross-modal relationships. In recent years, deep learning-based approaches have facilitated end-to-end multimodal fusion. For example, the authors of a previous study [4] proposed a two-stream architecture to extract deep audio and video features with affect-based loss. However, it did not explicitly model the correspondence between affective semantics and low-level representations. Subsequent studies [5,6] applied Canonical Correlation Analysis (CCA) for global feature projection; however, by neglecting the dynamic distinctions between matched and unmatched pairs, they limited the expressive capacity of the shared latent space. The CCDA model [7] advanced modality interactions through dual-attention fusion, but lacked the constraints to align the high-level semantic space with the low-level signal space, thereby reducing its generalization and applicability in consumer electronics scenarios.

In this work, to address these limitations, a dual-driven music–video matching framework that synergistically integrates perceptual and objective features is proposed. First, to mitigate distributional discrepancies between matched and unmatched pairs, a matching-state-adaptive Deep Canonical Correlation Analysis (DCCA) [8] module is introduced, which independently projects samples into tailored latent spaces for improved feature alignment. Second, fine-grained affective labels from music and video are incorporated as perceptual features and jointly modeled with objective representations to enrich cross-modal embedding. Finally, a multi-task network that incorporates self-attention and cross-modal mutual-attention mechanisms to capture temporal and spatial dependencies is designed. In addition, feature-consistency loss is introduced to align high-level semantics with low-level signals, thereby enhancing model robustness and generalization.

In summary, our main contributions are as follows:

(1) A dual-driven architecture that integrates fine-grained affective labels with audio–video features through coordinated modeling in both perceptual spaces and objective representations to enhance embedding quality and improve matching accuracy is proposed.
(2) A novel cross-modal dimensionality reduction method that explicitly distinguishes between matched and unmatched samples to optimize the projection spaces and substantially improve the expressiveness of cross-modal features is proposed.
(3) A multi-task learning framework that jointly employs self-attention, cross-modal mutual attention, and feature-consistency loss to effectively fuse music–video features while aligning semantic and signal spaces is proposed. Furthermore, an auxiliary affective regression task is incorporated to refine the primary matching prediction, thereby enhancing robustness and generalization.

The remainder of this paper is organized as follows. Section 2 reviews related work. Section 3 introduces the proposed method. Section 4 presents experimental results and analysis. Finally, Section 5 concludes the paper and discusses future research directions.

## 2. Related Work

This section reviews the chronological development of techniques for music–video affective matching. The primary challenge lies in constructing a unified cross-modal embedding space that faithfully aligns semantic information across auditory and visual modalities. Advances in this field can be broadly categorized into two dimensions: foundational feature representation learning and innovations in matching paradigms.

### 2.1. Multimodal Emotion Representation and Robust Affective Modeling

The efficacy of affectively matching music–video clips critically depends on the joint optimization of robust feature encoding and effective semantic-alignment strategies. Recent studies on robust multimodal emotion recognition have addressed challenges such as modality dropout and noise interference by integrating generative and discriminative models (e.g., RMER-DT [9]). The evolution of foundational feature learning can be delineated into three principal phases:

- Hand-Crafted Feature Engineering

Early studies relied on manually designed descriptors to capture modality-specific characteristics. In the audio domain, signal-processing features such as Mel-Frequency Cepstral Coefficients [10] and Linear Predictive Cepstral Coefficients [11] were employed to emulate aspects of human auditory perception. However, their dependence on expert heuristics limited their generalizability. In the visual domain, low-level image descriptors—including Histograms of Oriented Gradients [12], Local Binary Patterns [13], Haar-like features [14], and optical flow—were extracted to represent texture and motion. Yet, these features were insufficient for modeling the complex spatiotemporal dynamics inherent in video streams.

- Deep Feature Representation

With the rise in deep learning, end-to-end feature learning replaced manual engineering. In audio analysis, raw waveforms are typically converted into Mel spectrograms and processed using convolutional neural networks (e.g., VGGish [15] and YAMNet [16]) or Transformer-based architectures (e.g., AST [17] and GAFX [18]) to capture rich frequency-domain semantics. In parallel, video representation has advanced through 3D convolutional models (e.g., C3D [19]) and attention-based Transformers (e.g., TimeSformer [20], Swin Transformer [21], and S-ViT [22]), which effectively model hierarchical spatiotemporal patterns across multiple scales.

- Multimodal Representation Learning

Early efforts to map audio and visual modalities into a shared latent space relied on linear techniques such as Canonical Correlation Analysis (CCA), Partial Least Squares [23], and Bidirectional Latent Models [24]. However, their linear assumptions limited the ability to capture complex cross-modal relationships. To address this, nonlinear deep learning approaches were introduced. For example, DCCA employs multilayer perceptrons to learn nonlinear projections, while Adversarial Cross-Modal Retrieval [25] incorporates modality discriminators within a triplet-adversarial framework to reduce inter-modal distribution discrepancies more effectively. Contextual Interaction-Based Multimodal Emotion Analysis (CIME) [26] further emphasizes cross-modal semantic interactions and contextual dependencies, thereby enhancing affect recognition through enriched semantic representations.

### 2.2. Cross-Modal Alignment, Fusion, and Robust Matching Paradigms

Music–video matching techniques have evolved from simple distance-based measures on low-level features to advanced deep learning frameworks that explicitly model semantic alignment and modality invariance. Early approaches computed similarity directly between handcrafted audio and visual descriptors. For example, Shin et al. [1] represented each modality as a two-dimensional numerical sequence and applied distance metrics to achieve coarse audiovisual synchronization. Building on this, Surs et al. [27] proposed an unsupervised joint-embedding scheme, demonstrating its retrieval effectiveness on the large-scale YouTube-8M dataset [28].

To bridge the semantic gap, supervised deep-embedding methods were introduced. Zeng et al. [6,29] proposed a supervised CCA framework to project audio and video into a shared latent space, and they further improved retrieval accuracy by incorporating cluster-CCA refinement within a triplet-loss architecture. In parallel, Morgado et al. [30] employed cross-modal contrastive learning with an instance discrimination objective to reinforce semantic consistency across modalities. Subsequently, Wang et al. [31] further exploit contrastive learning to explicitly suppress noisy or negative cross-modal information, thereby enhancing robustness in multimodal emotion analysis. Beyond direct feature alignment, Xiang et al. [32] introduce generative text descriptions as intermediate semantic representations and apply contrastive learning to align the generated affective semantics with the original audiovisual features. Kang et al. [33] developed a supervised consistent feature learning framework with local group priors to align heterogeneous modalities in a discriminative shared space, which further enables effective retrieval even with partially unpaired data.

More recent advances have focused on improving robustness and discriminative power. For instance, the RAFT model [34] introduces adversarial fusion within a Transformer framework to enhance robustness against modality missingness and noisy inputs in multimodal sentiment analysis. Complementary to adversarial fusion strategies, the RMDG model [35] reconstructs missing modalities during training, thereby improving robustness in the presence of incomplete multimodal observations. The AML model [36] integrates modality-invariant representations with a robust cross-modal metric learning module in an end-to-end trainable framework, effectively mitigating heterogeneity in music–video matching. To address the scarcity of hard triplets and the resulting high-loss issue, Zeng et al. [37] introduced a semi-hard triplet training strategy that interpolates between low-loss anchors to generate challenging negative pairs, achieving an approximate 9.8% improvement in mean average precision on the AVE dataset. Most recently, Zeng et al. [38] proposed an Anchor-Aware Deep Metric Learning approach, which uncovers latent correlations among samples to further refine the structure and discriminability of the shared embedding space. In addition, several works (e.g., EMVAS [39]) focus on multimodal emotion visualization and interpretability, aiming to enhance transparency and human understanding of affective representations in multimodal systems.

While existing studies have explored cross-modal alignment, retrieval-based matching, or robustness through contrastive or adversarial objectives, most of them primarily rely solely on objective audiovisual features and model affect implicitly or as a secondary outcome of representation learning. In contrast, AFCLNet adopts an explicit dual-drive paradigm that jointly models objective audiovisual cues and perceptual affect representations. By integrating affect supervision as both an input signal and an auxiliary learning objective, our framework bridges low-level signal alignment and high-level emotional semantics. Furthermore, the proposed matching-state-adaptive DCCA and feature-consistency constraint enables robust modeling of both matched and mismatched cross-modal patterns, a capability that has received limited attention in prior fusion- and robustness-oriented approaches. As a result, AFCLNet is particularly well suited for fine-grained music–video affective matching under realistic conditions with incomplete perceptual annotations.

## 3. Materials and Methods

In this section, the overall framework of the proposed Attention and Feature-Consistency-Loss-Based Multi-Task Learning Network (AFCLNet) is first introduced. Subsequently, the matching-state-adaptive DCCA strategy for cross-modal dimensionality reduction is presented. Finally, the attention mechanisms and feature-consistency constraint within AFCLNet are detailed to illustrate the multi-level fusion and alignment process.

### 3.1. Framework Overview

**Figure 1:**
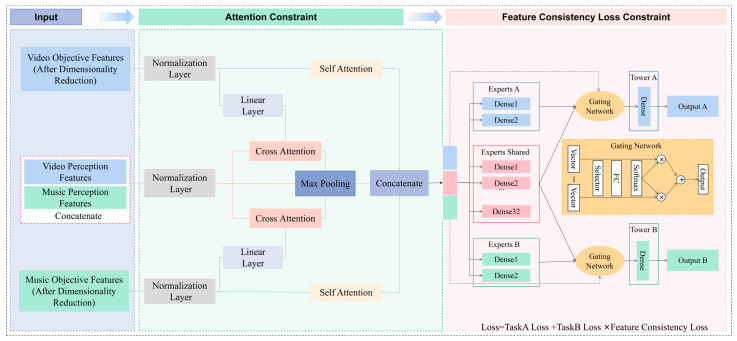
AFCLNet framework.

The overall architecture of the proposed AFCLNet is illustrated in Figure 1. The network processes two input streams: dimension-reduced objective features from both music and video, and fine-grained perceptual affect features corresponding to each modality. First, self-attention modules with eight attention heads are applied separately to the objective features of each modality to capture intra-modal dependencies. In parallel, a cross-modal mutual-attention module with four attention heads integrates perceptual and objective features, thereby modeling complementary inter-modal interactions. To ensure alignment between abstract semantic concepts and raw signal patterns, feature-consistency loss is introduced, thereby constraining the high-level semantic space to preserve fidelity with the corresponding low-level feature structures. During training, ground-truth affect labels provide reliable perceptual supervision. In inference, when manual annotations are unavailable, a pre-trained effect predictor trained solely on objective features automatically generates perceptual features. Finally, the network is optimized in a multi-task fashion, producing a scalar music–video matching score as the primary output and a 16-dimensional effect representation as an auxiliary output to refine semantic learning and enhance generalization.

### 3.2. Matching-State-Adaptive DCCA for Deep Cross-Modal Dimensionality Reduction

DCCA consists of two parallel deep neural networks, one for each modality, that teach nonlinear mappings to maximize inter-modal correlation. Specifically, each network transforms its input features through multiple nonlinear layers, after which canonical correlation analysis is applied to the network outputs to quantify their mutual dependence. During training, the negative correlation is minimized. This encourages the networks to produce maximally correlated embeddings.

However, conventional DCCA treats all sample pairs uniformly, overlooking the fact that matched and unmatched music–video pairs exhibit distinct cross-modal relationships. To address this limitation, a matching-state-adaptive DCCA module for cross-modal dimensionality reduction is introduced. Given a dataset of music–video pairs annotated on a 1–5 affective matching scale (Table 1), the training samples are partitioned into two subsets: matched pairs (score ≥ 3) and unmatched pairs (score < 3). Two independent DCCA models are then trained separately on each subset, learning state-specific cross-modal feature projections for the matched and unmatched conditions, respectively. This approach produces more discriminative and robust cross-modal embedding.

Each matching-state-adaptive DCCA model consists of two parallel multilayer perceptron (MLP) branches, corresponding to the music and video modalities, respectively. Each branch transforms its input features through an identical network architecture composed of an input layer matching the original feature dimensionality, followed by three hidden layers with 1024 neurons each, and an output layer that projects the features into a 40-dimensional latent space. Each hidden layer includes a fully connected linear transformation, a ReLU activation function, and a normalization layer to enhance training stability and nonlinear modeling capacity. This architecture provides sufficient representational power while maintaining a favorable balance between model complexity and computational efficiency.

**Figure 2:**
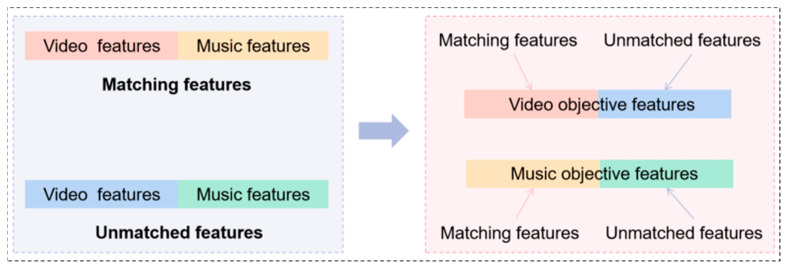
Music–video feature concatenation process.

In inference, both the matched DCCA and unmatched DCCA models are applied to each sample, generating two sets of dimension-reduced features for each sample: one corresponding to the matched state and the other to the unmatched state. For the audio modality, these matched and unmatched features are concatenated to form the final audio representation. The same process is applied to the video modality, producing the final video representation. This concatenation step is illustrated in Figure 2. Notably, both DCCA models are trained solely on the training set, ensuring that no test samples are introduced during model fitting. This safeguards against data leakage and upholds the fairness and generalizability of the evaluation.

**Figure 3:**
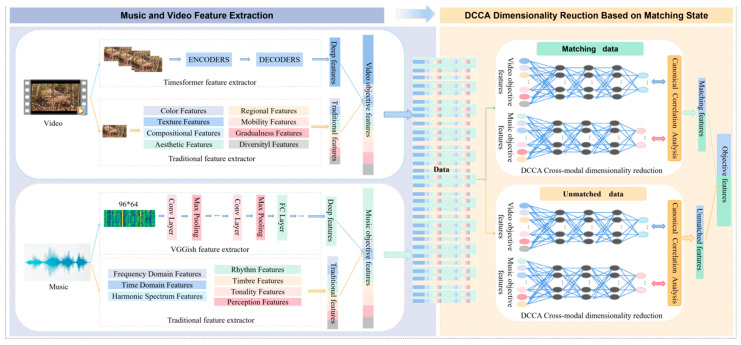
Feature extraction method and cross-modal dimensionality reduction framework based on matching-state-adaptive DCCA.

Before applying the matching-state-adaptive DCCA for dimensionality reduction, it is essential to first construct robust objective feature representations for both music and video. For the video modality, features are obtained by concatenating traditional handcrafted descriptors extracted from keyframes (e.g., color, texture, and compositional statistics) with deep representations generated by a pre-trained TimeSformer model. For the audio modality, features are similarly built by integrating conventional signal-processing metrics (e.g., time-domain, frequency-domain, and harmonic-spectrum descriptors) with deep embeddings extracted using a pre-trained VGGish network. These audio and video feature vectors are then provided as input to the matching-state-adaptive DCCA modules for cross-modal dimensionality reduction, as illustrated in Figure 3.

### 3.3. Attention Mechanism

The AFCLNet framework adopts a dual-drive multimodal fusion strategy that integrates perceptual and objective features using attention mechanisms. Specifically, a multi-head self-attention module is first applied independently to the objective features of each modality. In this process, the input features are first linearly projected to obtain the query, key, and value representations. These projected representations are then evenly split into multiple subspaces corresponding to different attention heads. Scaled dot-product attention is computed independently within each head. The resulting head-wise feature representations are subsequently concatenated along the feature dimension and passed through a linear projection to produce the final output. For the video modality, given an n-dimensional feature vector $x_{v}\in R^{n}$, the query $Q_{v}$, key $K_{v}$, and value $V_{v}$ are computed through learnable weight matrices $W_{Qv}$, $W_{Kv}$, $W_{Vv}\in R^{n\times n}$, respectively:

(1) $$Q_{v}=W_{Qv}x_{v},$$

(2) $$K_{v}=W_{Kv}x_{v},$$

(3) $$V_{v}=W_{Vv}x_{v},$$

where ${Q_{v},K_{v},V_{v}\in R}^{n}$. These are then divided into h parallel heads, each of dimension *d* = *n*/*h*:

(4) $$Q_{vi}={HeadSplit}_{i}(Q_{v})\in R^{d},$$

(5) $$K_{vi}={HeadSplit}_{i}(K_{v})\in R^{d},$$

(6) $$V_{vi}={HeadSplit}_{i}(V_{v})\in R^{d},$$

where ${HeadSplit}_{i}(∙)$ denotes the *i*-th segment. The scaled dot-product attention for head *i* is computed as follows:

(7) $${Attention}_{i}=softmax(\frac{Q_{vi}{K_{vi}}^{T}}{\sqrt{d}})V_{vi}\in R^{d},$$

where d is the dimensionality of the $K_{vi}$ vectors. The scaling by $\sqrt{d}$ follows the standard scaled dot-product attention mechanism and helps stabilize training by preventing extremely small gradients in high-dimensional spaces. Finally, the outputs of all heads are concatenated and projected through a linear transformation $W_{o}{\in R}^{n\times d}$, yielding the unified self-attention feature $x_{sv}$:

(8) $$x_{sv}=W_{o}[{Attention}_{1};\ldots ;{Attention}_{h}]\in R^{n},$$

where [;] denotes concatenation. The same procedure is applied to the audio objective features, yielding $x_{sa}\in R^{n}$.

To capture cross-modal interactions between objective and perceptual features, a multi-head mutual-attention module is introduced. Specifically, the raw video objective feature $x_{v}\in R^{n}$ is first projected into the same dimensional space as the perceptual feature $x_{p}\in R^{m}$, producing ${x_{v}}^{\prime }\in R^{m}$. These features are then fed into the mutual-attention block to compute the query $Q_{pv}$, key $K_{pv}$, and value $V_{pv}$:

(9) $$Q_{pv}=W_{Qpv}x_{p},$$

(10) $$K_{pv}=W_{Kpv}{x_{v}}^{\prime },$$

(11) $$V_{pv}=W_{Vpv}{x_{v}}^{\prime },$$

where $W_{Qpv},W_{Kpv}{,\;W}_{Vpv}\in R^{m\times m}$ are learnable projection matrices. After splitting into multiple heads, computing scaled dot-product attention, and concatenating the results, a video-guided perceptual embedding $x_{pv}\in R^{m}$ is obtained. An analogous operation is applied to the raw audio objective feature $x_{a}\in R^{n}$ and the perceptual feature $x_{p}\in R^{m}$ to obtain an audio-guided perceptual embedding $x_{pa}\in R^{m}$. The features $x_{p}$, $x_{pv}$, $x_{pa}$ are then concatenated, followed by max-pooling with a stride of 3 to distill the final perceptual feature vector $x_{cp}$. Finally, $x_{sv}$, $x_{sa}$, $x_{cp}$ are concatenated to construct the unified multimodal representation for downstream prediction.

### 3.4. Loss Functions

The AFCLNet framework formulates music–video clip matching score prediction as the primary task and 16-dimensional fused affect regression as an auxiliary task. Both are cast as regression problems, with mean absolute error (MAE) adopted as the loss criterion. The overall training objective is composed of three complementary loss terms, each serving a distinct role in the learning process. The music–video matching regression loss directly optimizes the primary task by minimizing the prediction error of matching scores. The fused 16-dimensional music–video effect regression loss is introduced as an auxiliary task, guiding the network to learn stable and semantically meaningful affect representations that enhance generalization across diverse audiovisual content. In addition, the feature-consistency loss enforces alignment between high-level semantic embeddings and low-level signal representations, thereby mitigating semantic drift across modalities. Through joint optimization, these loss components collaboratively improve matching accuracy while enhancing the robustness and generalizability of cross-modal representations. For a given sample $x_{i}$, let $y_{match}^{\left(i\right)}\in R$ denote the ground-truth matching score, and $y_{emotion}^{\left(i\right)}\in R^{16}$ is the ground-truth fused affect vector. The main task loss is defined as follows:

(12) $$L_{match}=\frac{1}{N}\sum_{i=1}^{N}\left|{\hat{y}}_{match}^{\left(i\right)}-y_{match}^{\left(i\right)}\right|,$$

where *N* denotes the batch size, ${\hat{y}}_{match}^{\left(i\right)}$ is the predicted matching score for input sample $x_{i}$, and $\left|∙\right|$ denotes the absolute value operation.

For the auxiliary task, a matching-state mask $m^{\left(i\right)}\in \left\{0,1\right\}$ is introduced, where $m^{\left(i\right)}=1$ for matched samples and $m^{\left(i\right)}=0$ otherwise, ensuring that only matched pairs contribute to the effect loss. The auxiliary effect loss is given by

(13) $$L_{emotion}=\frac{1}{N-k}\sum_{i=1}^{N}m^{\left(i\right)}{\left|\left|{\hat{y}}_{emotion}^{\left(i\right)}-y_{emotion}^{\left(i\right)}\right|\right|}_{1},$$

where *k* denotes the number of mismatched samples in batch *N*, and ${\hat{y}}_{emotion}^{\left(i\right)}$ represents the predicted 16-dimensional effect vector.

To enforce alignment between the high-level semantic embeddings and the low-level signal space, feature-consistency loss is introduced. Let $x_{sv}$ and $x_{sa}$ denote the self-attention-processed objective features for video and music, respectively. Their consistency is measured using the average squared $L_{2}$ distance over matched samples:

(14) $$L_{2}=\frac{1}{N-k}\sum_{i=1}^{N}m^{\left(i\right)}{\left|\left|x_{sv}^{\left(i\right)}-x_{sa}^{\left(i\right)}\right|\right|}_{2}^{2},$$

The overall training objective combines the three losses, with the feature-consistency term weighted relative to the auxiliary effect loss:

(15) $$L_{total}=L_{match}+L_{emotion}\times L_{2}.$$

## 4. Experiments

In this section, we first describe the datasets and implementation details. We then present comparative experiments against state-of-the-art methods to evaluate overall performance. Finally, a series of ablation studies is conducted to examine the individual contributions of dual-drive architecture, the matching-state-adaptive DCCA, the attention mechanisms, the feature-consistency constraint, and the emotion-guided auxiliary task.

### 4.1. Datasets

Through a systematic review of both domestic and international literature, existing datasets for music–video clip cross-modal matching can be broadly categorized into three groups:

(1) Affect-Similarity-Based Datasets: These datasets comprise separate audio and video subsets annotated with either continuous affect dimensions (e.g., the valence-arousal space) or discrete affect categories. A representative example is the Music–Video Emotion Dataset [40], in which two clips are considered matched if their affect scores are close or if they share the same categorical label.
(2) Self-Supervised Datasets: In this category, original video segments and their corresponding audio tracks naturally form positive pairs without requiring manual annotation. The MUVI dataset [41] exemplifies this approach, which is particularly effective for training cross-modal retrieval models.
(3) Regression-Oriented Matching Datasets: These datasets formulate music–video clip matching as a regression problem by assigning each pair a continuous matching score, typically ranging from 0 to 5. The Music–Video Matching Dataset [42] follows this paradigm, using fine-grained human annotations to capture varying degrees of audiovisual coherence, thereby providing a more nuanced representation of matching strength.

It is important to note that the first two types of datasets typically rely on binary matching decisions (i.e., matched vs. unmatched), thereby overlooking nuanced variations in matching strength—an aspect crucial for recommendation systems and user experience design. To address this limitation, the third category of datasets provides the foundation for our research, as it better captures multi-level matching relationships between music and video. The original dataset consists of 1000 music–video clip pairs, where positive pairs are directly extracted from movies or TV soundtracks, while negative pairs are constructed by replacing the original audio tracks. Each pair was evaluated by multiple annotators for audiovisual coherence, producing the ground-truth matching labels. To improve training efficacy and generalization, we expanded the dataset to 2200 pairs, following the methodology outlined in prior work. The 16-dimensional effect labels were obtained through subjective evaluation experiments. Each music–video clip was independently annotated by 10 human raters. After removing outliers, the final effect values were computed by averaging the remaining scores. This expansion enriched the sample distribution and increased the diversity of matching degrees.

### 4.2. Experimental Details

All experiments were implemented in TensorFlow and accelerated using an NVIDIA RTX 4090D GPU (NVIDIA Corporation, Santa Clara, CA, USA). Training was performed for 200 epochs with a batch size of 1024, using the Adam optimizer with an initial learning rate of 0.0001. An early-stopping strategy was applied, terminating training if the validation loss did not decrease for 30 consecutive epochs. The dataset was randomly split into training, validation, and test sets with a ratio of 8:1:1 using a fixed random seed of 42. All experiments were repeated 10 times with the same data partition strategy, and the reported results correspond to the average performance across runs to ensure statistical reliability. During training, ground-truth affect labels were used as perceptual inputs, whereas at inference, these labels were replaced with predictions generated by a LightGBM [43] model trained on the objective features, thereby simulating the absence of manual annotations in real-world deployments.

To evaluate the effectiveness of the proposed AFCLNet, its performance was compared with the following representative methods:

(1) CCA-GA-BPNN [44]: A Cluster-CCA-based feature fusion approach followed by a three-layer backpropagation neural network for matching score regression.
(2) VideoAdviser [45]: A multimodal transfer-learning framework that distills video-enhanced multimodal knowledge from a teacher model into modality-specific student models to improve matching accuracy.
(3) CCDA [7]: A dual-attention fusion framework that integrates semantic and representational features by combining self-attention and cross-attention mechanisms, optimized with a cross-correlation loss.
(4) This method applies CCA to fuse audio–video features, then employs XGBoost to predict two matching scores: x (derived from raw audiovisual features) and y (derived from affective semantic distances). The final score is obtained as a weighted sum of the two.
(5) Stacking: An ensemble stacking approach comprising four random forests and a support vector machine (SVM) as base learners, with an SVM serving as the meta-learner. This model follows the same input and label-availability scheme as our proposed method, i.e., real labels during training and LightGBM-predicted labels during inference.

Mean Absolute Error (MAE) and Mean Squared Error (MSE) are employed as performance metrics. MAE captures the average absolute deviation between predictions and ground-truth labels, reflecting overall regression accuracy. In contrast, MSE places greater emphasis on larger errors, thereby assessing the model’s sensitivity to outliers. For both metrics, lower values indicate better prediction performance. Together, these metrics provide a comprehensive evaluation of regression performance in the music–video clip matching task. To ensure fair comparison, methods originally designed for classification were adapted to regression tasks, while retrieval-oriented models were limited to their feature-extraction and fusion backbones. All models were trained and evaluated on our expanded music–video clip matching dataset.

### 4.3. Experimental Analysis

As shown in Table 2, the proposed AFCLNet achieved superior performance across both evaluation metrics, with an MAE of 0.109 and an MSE of 0.019, substantially outperforming all baseline methods. This confirms its effectiveness for music–video clip matching score prediction. By contrast, the traditional CCA-GA-BPNN model demonstrated limited feature-fusion capability, yielding an MAE of 0.178 and an MSE of 0.045. VideoAdviser and CCDA—two representative multimodal fusion approaches—improve accuracy through transfer learning and attention mechanisms, respectively, yet they still fall short of AFCLNet in terms of generalization. Notably, CCDA attained an MAE of 0.162 and an MSE of 0.041, indicating instability in modeling continuous matching distributions. In contrast, the Affective Similarity and CCA fusion method and the Stacking ensemble approach—which integrate affective semantics and ensemble strategies—achieved MAEs of 0.127 and 0.118 and MSEs of 0.027 and 0.021, respectively. These results highlight the positive effect of incorporating affective features on model performance. Ultimately, by jointly leveraging self-attention, cross-modal mutual attention, feature-consistency constraints, and a perceptual auxiliary task, AFCLNet achieved the best predictive performance. This demonstrates the effectiveness of multi-task learning and underscores the importance of coordinated modeling between perceptual and objective features for improved generalization.

### 4.4. Ablation Studies

To systematically evaluate the contribution of each key component in our framework, four groups of ablation studies were conducted on the expanded music–video clip matching dataset. All experiments were implemented in TensorFlow running on an NVIDIA RTX 4090D GPU. Performance was assessed using three metrics: MAE, MSE, and the coefficient of determination ($R^{2}$), where $R^{2}$ quantifies the proportion of variance explained by the model (with values closer to 1 indicating a better fit). The specific study designs are as follows:

(1) Dual-Drive Architecture: With the cross-modal dimensionality reduction strategy fixed, three input configurations were evaluated: (i) perceptual features only, (ii) objective features only, and (iii) a combination of perceptual and objective features. It is worth noting that when only a single feature type is used, the attention modules cannot operate as intended.
(2) Cross-Modal Dimensionality Reduction Strategy: With the AFCLNet backbone fixed, four dimensionality reduction schemes were compared: no dimensionality reduction, standard DCCA, cluster-increased DCCA (C-DCCA), and matching-state-adaptive DCCA.
(3) Joint Constraint Mechanisms: With input features fixed, five network variants were evaluated: (i) a base CGC network without any attention or alignment constraints, (ii) the base CGC network with self-attention, (iii) the base CGC network with cross-attention, (iv) the base CGC network with feature-consistency loss, and (v) the base CGC network with combined self-attention, cross-attention, and feature-consistency constraints (corresponding to our proposed AFCLNet).
(4) Auxiliary Task Design: Using the same input features and backbone, three auxiliary tasks were compared: (i) voice-presence detection, (ii) person-presence recognition, and (iii) 16-dimensional affect-fusion regression.

The results of these ablation studies are summarized in Table 3, Table 4, Table 5 and Table 6.

As shown in Table 3, when only fine-grained perceptual features were provided, the model failed to capture low-level music–video correlations, yielding an MAE of 0.144, an MSE of 0.031, and an $R^{2}$ value of 0.311. In contrast, using only objective features reduced via matching-state-adaptive DCCA led to improved performance (MAE = 0.112, MSE = 0.022, $R^{2}$ = 0.588), suggesting that objective representations more effectively encode audiovisual correspondence. The best results were obtained when combining perceptual features with matching-state-adaptive DCCA-reduced objective features (MAE = 0.109, MSE = 0.018, $R^{2}$ = 0.623), demonstrating the complementary strengths of dual-drive modeling: perceptual features capture high-level affective semantics, while objective features preserve low-level physical signals. Their joint representation bridges the semantic-signal “cognitive gap,” significantly enhancing embedding quality and prediction accuracy.

Table 4 reports the comparison of four cross-modal dimensionality reduction strategies. Direct concatenation of raw objective features led to redundancy and modality distribution mismatches, resulting in degraded performance (MAE = 0.170, MSE = 0.042, $R^{2}$ = 0.100). Standard DCCA alleviated these issues by improving alignment (MAE = 0.157, MSE = 0.038, $R^{2}$ = 0.196), demonstrating its effectiveness for feature fusion. C-DCCA yielded a slight improvement in MAE (0.167) but produced a lower $R^{2}$ value (0.091), suggesting that clustering constraints may conflict with the matching objective. In contrast, matching-state-adaptive DCCA, which explicitly distinguishes between matched and unmatched pairs during projection, achieved the best results (MAE = 0.109, MSE = 0.018, $R^{2}$ = 0.623), confirming its effectiveness for cross-modal feature alignment in music–video clip matching.

Table 5 evaluates the contributions of attention and alignment constraints. The base CGC network achieved an $R^{2}$ value of 0.505, reflecting its basic modeling capacity. Incorporating self-attention increased $R^{2}$ to 0.566 by capturing intra-modal dependencies, and adding cross-attention further improved $R^{2}$ to 0.584 through enhanced inter-modal interactions. Introducing feature-consistency loss alone yielded an $R^{2}$ value of 0.580 by aligning high-level semantics with low-level signals. The full AFCLNet configuration—combining self-attention, cross-attention, and feature-consistency constraints—achieved the best overall performance (MAE = 0.109, MSE = 0.018, $R^{2}$ = 0.623), demonstrating strong synergistic effects and improved robustness.

Table 6 assesses the impact of different auxiliary tasks. Voice-presence detection and person-presence recognition yielded only modest improvements ($R^{2}$ = 0.509 and 0.494, respectively), indicating limited transferability from weakly correlated tasks. In contrast, the 16-dimensional effect-fusion regression auxiliary task substantially enhanced performance (MAE = 0.109, MSE = 0.018, $R^{2}$ = 0.623), demonstrating that effective-semantic guidance promotes deeper cross-modal synergy, thereby improving generalization and prediction accuracy.

Overall, the ablation studies confirmed that each component contributed meaningfully to the strong performance and robustness of the proposed music–video clip matching framework.

### 4.5. Qualitative Visualization

To further assess the interpretability and effectiveness of the model, a qualitative case analysis was conducted on four representative music–video pairs randomly selected from the test set: two matched pairs and two mismatched pairs.

Table 7 reports the predicted and ground-truth matching scores for these cases. In both scenarios, the model’s predictions closely aligned with the reference values, demonstrating its effectiveness and robustness in distinguishing between semantically coherent and incoherent music–video pairs.

**Figure 4:**
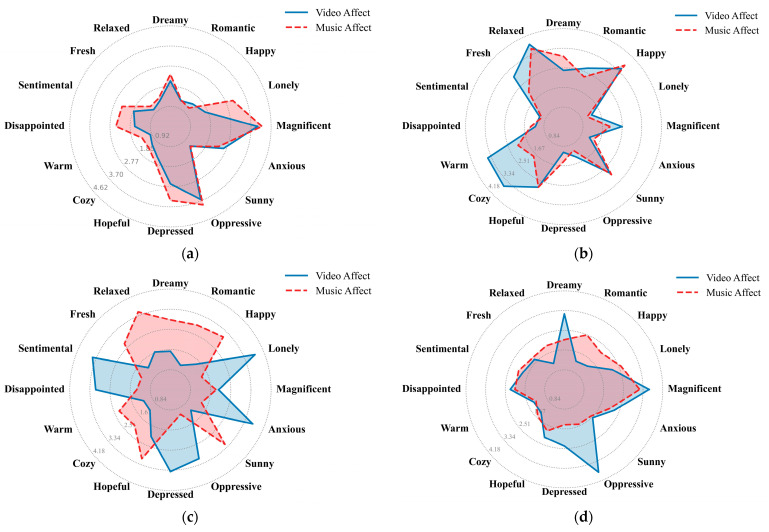
Radar plots depicting the 16-dimensional emotion distributions of music and video modalities for (**a**) matched pair 1; (**b**) matched pair 2; (**c**) mismatched pair 1; and (**d**) mismatched pair 2.

Figure 4 illustrates the 16-dimensional emotional distributions of music and video across different cases. The matched pair 1 exhibits strong affective consistency across modalities, while matched pair 2 shows partial discrepancies in emotional dimensions. In contrast, mismatched pair 1 displays clear divergences in emotional characteristics, whereas mismatched pair 2 presents partial emotional overlaps. These results indicate that our proposed model can effectively handle not only cases of clear emotional similarity or dissimilarity, but also those with subtle affective differences where partial congruence still leads to correct matching or rejection decisions.

**Figure 5:**
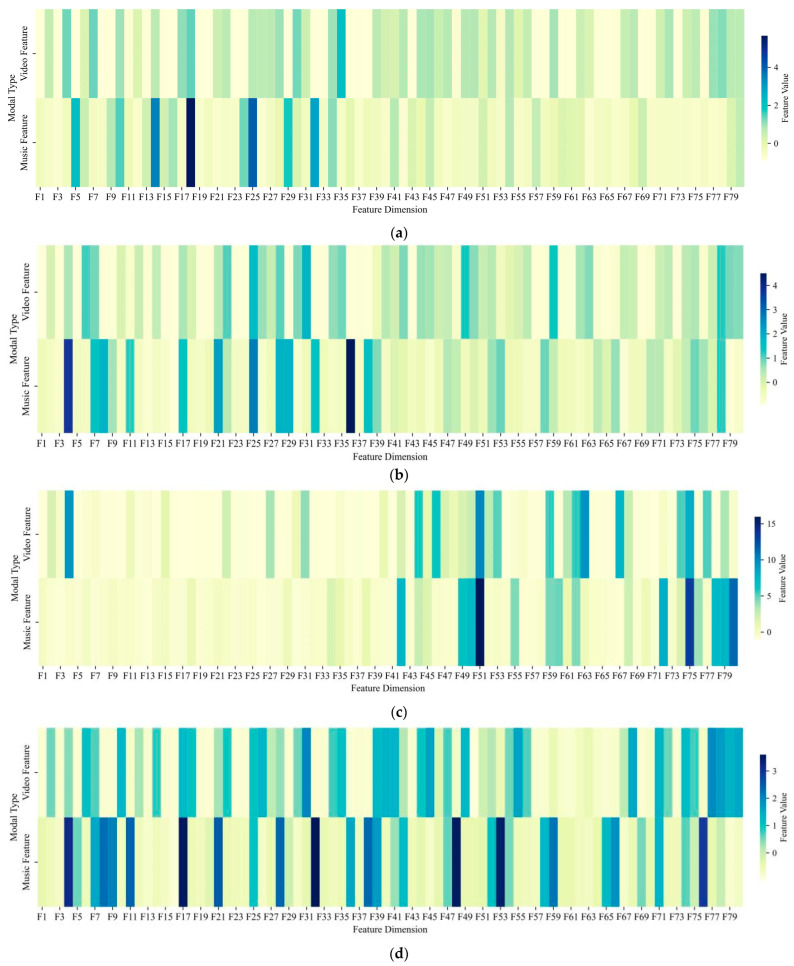
Heatmaps of projected feature embedding obtained via matching-state-adaptive DCCA: (**a**) matched pair 1; (**b**) matched pair 2; (**c**) mismatched pair 1; and (**d**) mismatched pair 2.

Figure 5 presents heatmaps of cross-modal feature activations after projection through the matching-state-adaptive DCCA. For the matched pairs, most corresponding dimensions exhibit highly consistent activation levels, indicating strong cross-modal coherence. Nevertheless, certain dimensions display localized discrepancies, suggesting subtle modality-specific emotional expressions. In contrast, the mismatched pairs showed pronounced inversions in activation patterns across several principal dimensions, reflecting divergent feature representations between modalities. These qualitative analyses reinforce the quantitative findings, confirming the effectiveness of the dual-drive framework in integrating fine-grained emotional features with objective representations and underscoring the critical role of matching-state-adaptive DCCA in achieving enhanced cross-modal alignment.

## 5. Conclusions

This paper has presented a novel multi-task learning framework from the perspective of emotional correlation and integration, which significantly improves the accuracy of music–video clip matching with complex effects. In the proposed approach, fine-grained affective labels are first extracted from music and video segments and used as perceptual features. These perceptual features are then integrated with both handcrafted and deep objective features to form a robust basis for cross-modal fusion. To further improve feature representation, matched and unmatched samples are decoupled and projected through separate DCCA mappings, enabling more discriminative low-dimensional subspace learning. Finally, self-attention and mutual-attention modules are introduced to capture intra-modal and inter-modal correlations between perceptual features and reduced objective features, while feature-consistency loss enforces alignment between high-level semantic abstractions and low-level signal representations. In addition, an auxiliary affect-regression task strengthens generalization and further improves matching score prediction. Experimental results confirm that the proposed framework achieves state-of-the-art performance, substantially surpassing existing baselines.

Despite the strong representational power achieved through the combination of handcrafted and deep features, generalizing to large-scale and diverse datasets remains challenging. Future work will explore the integration of large-scale multimodal pre-trained models to strengthen universal audio–video semantic representations, thereby enhancing adaptability and robustness in open-domain, real-world scenarios.

## Figures and Tables

**Table 1:** Five-Level Matching Score Scale.

Score	Matching Degree Labeling
1	Completely mismatched
2	Do not match well
3	Relatively matched
4	Strong match
5	Complete match

**Table 2:** Comparative Algorithm Performance.

Model	MAE	MSE
Modified CCA-GA-BPNN	0.178	0.045
Modified VideoAdviser	0.179	0.047
Modified CCDA	0.162	0.041
Affective similarity and CCA feature fusion	0.127	0.027
Stacking	0.118	0.021
AFCLNet (ours)	0.109	0.018

**Table 3:** Ablation Results for the Dual-Drive Architecture.

Input	MAE	MSE	$R^{2}$
Perceptual features only	0.144	0.031	0.311
Objective features only	0.112	0.022	0.588
Perceptual and objective features (ours)	0.109	0.018	0.623

**Table 4:** Ablation Results for the Cross-Modal Dimensionality Reduction Strategy.

Input	MAE	MSE	$R^{2}$
No dimensionality reduction	0.170	0.042	0.100
Objective features reduced via standard DCCA	0.157	0.038	0.196
Objective features reduced via C-DCCA	0.167	0.043	0.091
Objective features reduced via matching-state-adaptive DCCA (ours)	0.109	0.018	0.623

**Table 5:** Ablation Results for the Joint Constraint Mechanisms.

Input	MAE	MSE	$R^{2}$
Base CGC network	0.125	0.023	0.505
Base CGC network with self-attention	0.115	0.029	0.566
Base CGC network with cross-attention	0.112	0.028	0.584
Base CGC network with feature-consistency loss	0.114	0.026	0.580
AFCLNet (ours)	0.109	0.018	0.623

**Table 6:** Ablation Results for the Auxiliary Task Design.

Input	MAE	MSE	$R^{2}$
Voice-presence detection	0.118	0.022	0.509
Person-presence detection	0.119	0.023	0.494
16-Dimensional fused affect regression (ours)	0.109	0.018	0.623

**Table 7:** Ground truth and predicted matching scores of two representative video–music pairs.

Samples	Video and Music Clips	Affective Matching Values	
		Subjective Value	Predicted Value
Matched pair 1	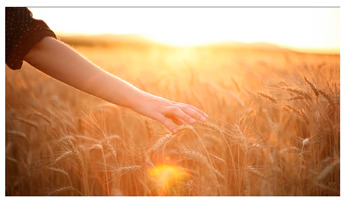	4.40	4.25
	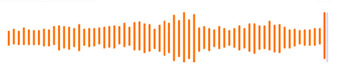		
Matched pair 2	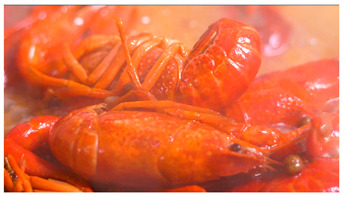	3.60	3.88
	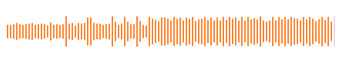		
Mismatched pair 1	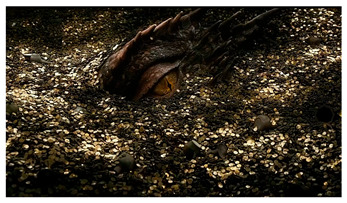	2.80	2.95
	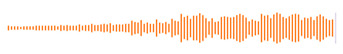		
Mismatched pair 2	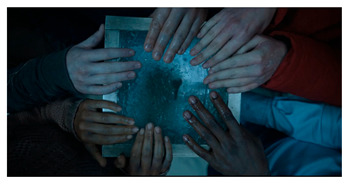	1.80	1.74
	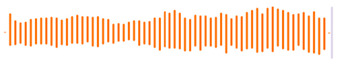		

## Data Availability

The raw data supporting the conclusions of this article will be made available by the authors on request.

## References

[1] Shin K.-H., Lee I.-K. Music synchronization with video using emotion similarity. Proceedings of the 2017 IEEE International Conference on Big Data and Smart Computing (BigComp).

[2] Chao J., Wang H., Zhou W., Zhang W., Yu Y. Tunesensor: A semantic-driven music recommendation service for digital photo albums. Proceedings of the China Semantic Web Symposium.

[3] Wu X., Qiao Y., Wang X., Tang X. (2016). Bridging music and image via cross-modal ranking analysis. IEEE Trans. Multimed..

[4] Li B., Kumar A. Query by Video: Cross-modal music retrieval. Proceedings of the International Society for Music Information Retrieval Conference.

[5] Shao J., Wang L., Zhao Z., Su F., Cai A. (2016). Deep canonical correlation analysis with progressive and hypergraph learning for cross-modal retrieval. Neurocomputing.

[6] Zeng D., Yu Y., Oyama K. Audio-visual embedding for cross-modal music video retrieval through supervised deep CCA. Proceedings of the 2018 IEEE International Symposium on Multimedia (ISM).

[7] Wang P., Liu S., Chen J. (2024). CCDA: A novel method to explore the cross-correlation in dual-attention for multimodal sentiment analysis. Appl. Sci..

[8] Andrew G., Arora R., Bilmes J., Livescu K. Deep canonical correlation analysis. Proceedings of the International Conference on Machine Learning.

[9] Zhu X., Wang Y., Cambria E., Rida I., López J.S., Cui L., Wang R. (2025). RMER-DT: Robust multimodal emotion recognition in conversational contexts based on diffusion and transformers. Inf. Fusion.

[10] Davis S., Mermelstein P. (1980). Comparison of parametric representations for monosyllabic word recognition in continuously spoken sentences. IEEE Trans. Acoust. Speech Signal Process..

[11] Atal B.S. (1976). Automatic recognition of speakers from their voices. Proc. IEEE.

[12] Dalal N., Triggs B. Histograms of oriented gradients for human detection. Proceedings of the IEEE Computer Society Conference on Computer Vision and Pattern Recognition (CVPR).

[13] Ojala T., Pietikainen M., Maenpaa T. (2002). Multiresolution gray-scale and rotation invariant texture classification with local binary patterns. IEEE Trans. Pattern Anal. Mach. Intell..

[14] Papageorgiou C.P., Oren M., Poggio T. A general framework for object detection. Proceedings of the International Conference on Computer Vision (ICCV).

[15] Hershey S., Chaudhuri S., Ellis D.P.W., Gemmeke J.F., Jansen A., Moore R.C., Plakal M., Platt D., Saurous R.A., Seybold B. CNN architectures for large-scale audio classification. Proceedings of the 2017 IEEE International Conference on Acoustics, Speech and Signal Processing (ICASSP).

[16] Ellis M. YAMNet: AudioSet Based YAMNet. GitHub Repository, 2017. https://www.tensorflow.org/hub/tutorials/yamnet?hl=zh-cn.

[17] Gong Y., Chung Y.-A., Glass J. AST: Audio spectrogram transformer. Proceedings of the Interspeech.

[18] Bu Z., Zhang H., Zhu X. (2022). GAFX: A general audio feature extractor. arXiv.

[19] Tran D., Bourdev L., Fergus R., Torresani L., Paluri M. Learning spatiotemporal features with 3D convolutional networks. Proceedings of the IEEE International Conference on Computer Vision (ICCV).

[20] Bertasius G., Wang H., Torresani L. Is space-time attention all you need for video understanding?. Proceedings of the International Conference on Machine Learning.

[21] Liu Z., Ning J., Cao Y., Wei Y., Zhang Z., Lin S., Hu H., Microsoft Research Asia, University of Science and Technology of China, Huazhong University of Science and Technology Video swin transformer. Proceedings of the IEEE/CVF Conference on Computer Vision and Pattern Recognition (CVPR).

[22] Zhao Y., Luo C., Tang C., Chen D., Codella N., Zha Z.-J. Streaming video model. Proceedings of the IEEE/CVF Conference on Computer Vision and Pattern Recognition (CVPR).

[23] Rosipal R., Krämer N. (2006). Overview and recent advances in partial least squares. Subspace, Latent Structure and Feature Selection, Proceedings of the Statistical and Optimization Perspectives Workshop, SLSFS 2005, Bohinj, Slovenia, 23–25 February 2005.

[24] Sharma A., Kumar A., Daume H., Jacobs D.W. Generalized Multiview Analysis: A discriminative latent space. Proceedings of the IEEE Conference on Computer Vision and Pattern Recognition (CVPR).

[25] Wang B., Yang Y., Xu X., Hanjalic A., Shen H.T. Adversarial cross-modal retrieval. Proceedings of the ACM International Conference on Multimedia.

[26] Wang R., Guo C., Shabaz M., Rida I., Cambria E., Zhu X. (2025). CIME: Contextual Interaction-Based Multimodal Emotion Analysis with Enhanced Semantic Information. IEEE Trans. Comput. Soc. Syst..

[27] Surs D., Duarte A., Salvador A., Torres J., Giro-i-Nieto X. Cross-modal embeddings for video and audio retrieval. Proceedings of the European Conference on Computer Vision (ECCV) Workshops.

[28] Abu-El-Haija S., Kothari N., Lee J., Natsev P., Toderici G., Varadarajan B., Vijayanarasimhan S. (2016). YouTube-8M: A large-scale video classification benchmark. arXiv.

[29] Zeng D., Yu Y., Oyama K. (2020). Deep triplet neural networks with cluster-CCA for audio-visual cross-modal retrieval. ACM Trans. Multimed. Comput. Commun. Appl..

[30] Morgado P., Vasconcelos N., Misra I. Audio-visual instance discrimination with cross-modal agreement. Proceedings of the IEEE/CVF Conference on Computer Vision and Pattern Recognition (CVPR).

[31] Wang R., Wang Y., Cambria E., Fan X., Yu X., Huang Y., E X., Zhu X. (2025). Contrastive-Based Removal of Negative Information in Multimodal Emotion Analysis. Cogn. Comput..

[32] Xiang J., Zhu X., Cambria E. (2025). Integrating audio–visual text generation with contrastive learning for enhanced multimodal emotion analysis. Inf. Fusion.

[33] Kang C., Xiang S., Liao S., Xu C., Pan C. (2015). Learning Consistent Feature Representation for Cross-Modal Multimedia Retrieval. IEEE Trans. Multimed..

[34] Wang R., Xu D., Cascone L., Wang Y., Chen H., Zheng J., Zhu X. (2025). RAFT: Robust Adversarial Fusion Transformer for multimodal sentiment analysis. Array.

[35] Zhang Y., Chen H., Rida I., Zhu X. (2025). A Generative Random Modality Dropout Framework for Robust Multimodal Emotion Recognition. IEEE Intell. Syst..

[36] Zheng A., Hu M., Jiang B., Yu Y. (2022). Adversarial-metric learning for audio-visual cross-modal matching. IEEE Trans. Multimed..

[37] Zeng D., Ikeda K. (2023). Two-stage triplet loss training with curriculum augmentation for audio-visual retrieval. arXiv.

[38] Zeng D., Wang Y., Ikeda K. Anchor-aware deep metric learning for audio-visual retrieval. Proceedings of the International Conference on Multimedia Retrieval.

[39] Zhu X., Feng H., Cambria E., Huang Y., Ju M., Yuan H. (2025). EMVAS: End-to-end multimodal emotion visualization analysis system. Complex Intell. Syst..

[40] Pandeya Y.R., Lee J. (2021). Deep learning-based late fusion of multimodal information for emotion classification of music video. Multimed. Tools Appl..

[41] Chua P., Makris D., Herremans D., Roig G., Agres K. (2022). Predicting emotion from music videos: Exploring the relative contribution of visual and auditory information to affective responses. arXiv.

[42] Su Z., Feng Y., Liu J., Peng J., Jiang W., Liu J. (2024). An audiovisual correlation matching method based on fine-grained emotion and feature fusion. Sensors.

[43] Ke G., Meng Q., Finley T., Wang T., Chen W., Ma W., Ye Q., Liu T.-Y. LightGBM: A highly efficient gradient boosting decision tree. Proceedings of the International Conference on Neural Information Processing Systems.

[44] Zhao Y. (2020). Research on Stock Price Prediction Model Based on CCA-GA-BPNN Comprehensive Technology. Master’s Thesis.

[45] Wang Y., Zeng D., Wada S., Ikeda K. (2023). VideoAdviser: Video knowledge distillation for multimodal transfer learning. IEEE Access.

